# Integrative Metabolome and Transcriptome Analyses Provide Insights into Carotenoid Variation in Different-Colored Peppers

**DOI:** 10.3390/ijms242316563

**Published:** 2023-11-21

**Authors:** Junheng Lv, Ruihao Zhang, Yunrong Mo, Huidan Zhou, Mengjuan Li, Rui Wu, Hong Cheng, Mingxian Zhang, Huasu Wang, Wei Hua, Qiaoling Deng, Kai Zhao, Minghua Deng

**Affiliations:** 1 Key Laboratory of Vegetable Biology of Yunnan Province, College of Landscape and Horticulture, Yunnan Agricultural University, Kunming 650201, China; 2020061@ynau.edu.cn (J.L.); zrh@yaas.org.cn (R.Z.); moyunrong@126.com (Y.M.); zhd1025@163.com (H.Z.); 13150515440@163.com (M.L.); wr1236542020@163.com (R.W.); chengh0222@163.com (H.C.); zmx11234566@163.com (M.Z.); hw131211@163.com (W.H.); dql0704@163.com (Q.D.); 2 Horticulture Research Institute, Yunnan Academy of Agricultural Sciences, Kunming 650205, China

**Keywords:** pepper, carotenoid, color formation, metabolome, transcriptome

## Abstract

Carotenoids are important pigments in pepper fruits. The colors of each pepper are mainly determined by the composition and content of carotenoid. The ‘ZY’ variety, which has yellow fruit, is a natural mutant derived from a branch mutant of ‘ZR’ with different colors. ZY and ZR exhibit obvious differences in fruit color, but no other obvious differences in other traits. To investigate the main reasons for the formation of different colored pepper fruits, transcriptome and metabolome analyses were performed in three developmental stages (S1–S3) in two cultivars. The results revealed that these structural genes (*PSY1*, *CRTISO*, *CCD1*, *CYP97C1*, *VDE1*, *CCS*, *NCED1* and *NCED2*) related to carotenoid biosynthesis were expressed differentially in the two cultivars. Capsanthin and capsorubin mainly accumulated in ZR and were almost non-existent in ZY. S2 is the fruit color-changing stage; this may be a critical period for the development of different color formation of ZY and ZR. A combination of transcriptome and metabolome analyses indicated that *CCS*, *NCED2*, *AAO4*, *VDE1* and *CYP97C1* genes were key to the differences in the total carotenoid content. These new insights into pepper fruit coloration may help to improve fruit breeding strategies.

## 1. Introduction

Peppers, which are widely cultivated worldwide, are among the most important crop of the Solanaceae family, coming second only to tomatoes in terms of production and consumption. Carotenoids are continuously synthesized and accumulated during the ripening stage of pepper fruits, and over 40 types of carotenoids have been identified in peppers [1,2]. The color of chili pepper fruits is highly correlated with their visual appeal and marketability and can also serve as an important indicator of ripeness. Carotenoids are important secondary metabolites in plants, and carotenoids in pepper fruits have both health-related and economic significance. In addition, carotenoids are precursors of volatile compounds, such as beta-ionone, as well as the hormones abscisic acid (ABA) and monocolactone, which are involved in various plant growth and development processes [3]. Also taken as the precursors and antioxidants of vitamin A, carotenoids play a vital role in human health. For example, β-cryptoxanthin was the precursor of vitamin A and zeaxanthin; its high antioxidant activity protects the eyes from disease, as well as from certain cancers [3]. 

Research on the biosynthesis pathway of horticultural plant carotenoids is well established [4]. GGPP serves as the direct precursor substance for the synthesis of carotenoids [5]. Through the action of phytoene synthase (PSY), the colorless phytoene is produced by condensing GGPP. This step is critical to carotenoid synthesis, with *PSY* serving as the main rate-limiting enzyme in the process [6,7]. Under the catalysis of two dehydrogenases and two isomerases with similar structures and functions, hydrogenated lycopene generates red lycopene [8]. After the synthesis of lycopene, the formation of carotenoids diverges into two important branches. One branch synthesizes alpha-carotene and lutein, while the other transforms lycopene into various pigments such as lutein, zeaxanthin, capsanthin, capsorubin, and neoxanthin under different enzyme catalysis [9,10,11]. Due to differences in the biosynthesis and regulation of carotenoids in pepper fruits, the composition and content of carotenoid components in peppers of different colors vary [12]. Carotenoids, the most important pigments in pepper fruits, have widely differing content among fruits of different colors. For example, the content of carotenoids in red pepper fruits can reach 163mg/kg, while it reaches a maximum of 28mg/kg in yellow fruits [13]. Capsanthin is the major carotenoid component in red pepper fruits, accounting for 46% of the total carotenoids. In orange pepper fruits, zeaxanthin is the major pigment component, accounting for 16% of the total carotenoids, while yellow pepper fruits accumulate more lutein, zeaxanthin, and cryptoxanthin components [14].

Changing the expression of key genes in the biosynthesis or degradation pathways of carotenoids can influence their accumulation. Studies have shown that simultaneously overexpressing the *DXS*, *PSY1*, and *CRTI* genes in rice endosperm can significantly increase the total carotenoid content [15]. Inducing genotype-specific expression and functional-specific overexpression of the *PSY* gene can effectively enhance the carotenoid content in non-green tissues of plants, making it the preferred gene for improving plant carotenoids through genetic engineering [7,16]. Through silencing one or multiple genes, studies have discovered that *CCS*, *PSY*, *LCY-B*, and *CRT-Z* are key to the biosynthetic pathway of carotenoids in peppers. Their diverse expression patterns have resulted in the rich and varied colors of pepper fruits [17,18]. Transcription factors can directly or indirectly regulate key genes in the carotenoid biosynthesis pathway, leading to metabolic changes [19]. In *Arabidopsis*, *PIF1* inhibits the expression of *PSY* by binding to its promoter [20]. In citrus, *CsMADS5* positively regulates fruit carotenoid biosynthesis by directly binding to the promoters of *PSY*, *PDS*, and *LCYb1* [21]. In tomato, many transcription factors have been found to be involved in the regulation of carotenoid biosynthesis [22]. For instance, *SlSGR1* and *RIN* can directly bind to *SlPSY1*, thus affecting carotenoid biosynthesis [23,24]. Overexpression of *SlWRKY35* increases carotenoid accumulation, and targeting *SlWRKY35* can enhance lutein production in tomato fruit [25]. Overexpression of *SlBBX20* leads to significant accumulation of carotenoids and flavonoids in tomato fruit [26]. Mutation of the *SlIDI1* gene reduces the expression of *SlBCH1*, which is beneficial for β-carotene accumulation, resulting in unique orange-colored flesh [27]. In addition to transcription factor-mediated transcriptional regulation, post-transcriptional, post-translational, and epigenetic regulation also play a role in the metabolism of carotenoids.

The ‘ZY’ variety, a natural mutant derived from a branch mutant of ‘ZR’, was found in 2019 in Kunming County, Yunnan Province, China. The peel of the ZY, which was yellow instead of red, made it clearly distinguishable from the ZR. There is no significant difference between ZY and ZR in any traits except color difference, and its characteristics have proven stable after years of observation and evaluation. However, the molecular mechanism of color variation in this mutant has not been comprehensively studied. ZY and ZR are excellent materials for studying the formation mechanism of differences in carotenoid synthesis and accumulation in different-colored peppers. In this study, high-performance liquid chromatography (HPLC) was used to identify the composition and content of carotenoids in the pepper fruits of ZY and ZR. Furthermore, RNA-seq was used to delve into the molecular mechanisms underlying carotenoid differences between the two varieties. By combining transcriptome and metabolome analyses, this study could provide a new insight into the biosynthesis of carotenoids in pepper fruits.

## 2. Results

### 2.1. Dynamic Changes in the Transcriptome between ZY and ZR

**Figure 1:**
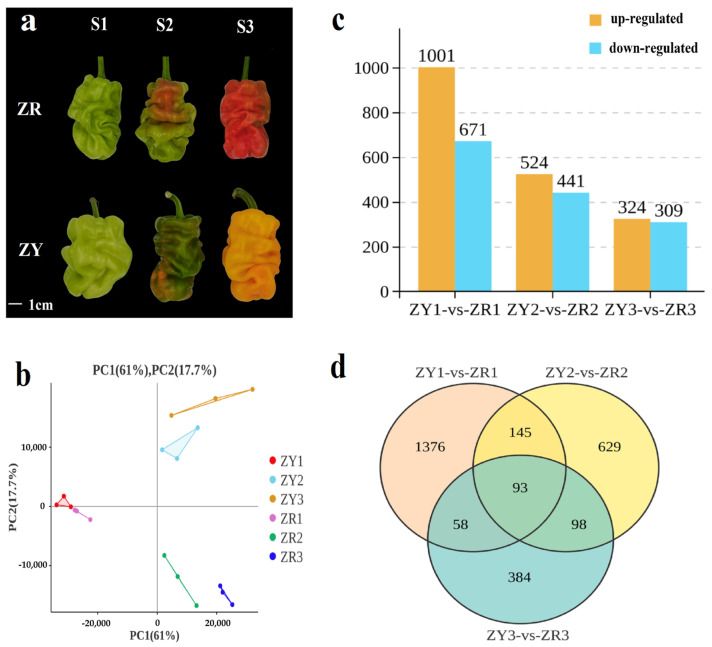
Fruit colors and number of DEGs between two pepper varieties. (**a**) Changes in the color of pepper fruits from the two cultivars in the three developmental stages; (**b**) PCA score plot; (**c**) The number of upregulated and downregulated DEGs in the pepper at three developmental stages; (**d**) Venn diagram of DEGs in different comparison groups.

To investigate the potential mechanism underlying the differences in carotenoid accumulation between red and yellow pepper fruit (Figure 1a), we analyzed the differentially expressed genes (DEGs) using principal component analysis. We analyzed the gene expression levels of multiple samples from each organism and found that the differences between red and yellow pepper fruit were most pronounced during developmental stages S2 and S3 (Figure 1b). When comparing samples at the same stage between ZY and ZR, we found 1672 DEGs (1001 upregulated and 671 downregulated) (Table S1), 965 DEGs (524 upregulated and 441 downregulated) (Table S2), and 633 DEGs (324 upregulated and 309 downregulated) (Table S3) in the S1, S2, and S3 stages, respectively (Figure 1c). The differences were most pronounced in the S1 stage, and the DEGs in all three stages were more upregulated in ZY. A total of 93 genes were differentially expressed in both ZY and ZR (Figure 1d). As the development progressed over time, the number of DEGs specific to each stage gradually decreased, as follows: S1 (1376), S2 (629), and S3 (384) (Figure 1d).

### 2.2. Gene Expression Patterns of the Carotenoid Pathway

Analysis of genes related to carotenoid metabolism pathways revealed that structural genes with differential expression varied between ZY and ZR samples in different stages. In the S1 stage, only the limiting enzyme *NCED2* (*Capana01g003704*) controlling carotenoid conversion to ABA showed differential expression, with 2.3-fold higher expression in ZR than in ZY. In the S2 stage, both *CCS* (*Capana06g000615*) and *NCED2* showed significantly higher expression in ZR compared to ZY, with CCS upregulated 9.4-fold in ZR. Meanwhile, *CRTISO* (*Capana11g002179*), *VDE1* (*Capana12g001449*) and *CCD1* (*Capana01g003704*) showed significantly lower expression in ZR compared to ZY. In S3 stage, *NCED1* (*Capana00g003114*) and *CCS* showed high expression in ZR, with *CCS* exhibiting 10-fold higher expression in ZR compared to ZY, while the key limiting gene *PSY1* (*Capana02g002284*) for carotenoid synthesis showed approximately 2.9-fold higher expression in ZY compared to ZR. The differential gene results infer that *CCS* is the most significant contributor to the different colors observed in ZR and ZR fruits.

DEG analysis revealed that the number of differentially expressed structural genes in the carotenoid synthesis pathway was relatively low at the same stage between ZR and ZY. Therefore, we further analyzed transcription factor genes among the DEGs. The results showed that many transcription factors were significantly differentially expressed at different stages, including the transcription factor families of bHLH, MYB, ERF, WRKY, and NAC. At the S1 stage, the largest number of differentially expressed transcription factors was identified, including 20 ERF, 12 WRKY, 12 MYB, 9 bHLH, and 9 NAC genes (Table S4).

### 2.3. Functional Enrichment Analysis of DEGs

**Figure 2:**
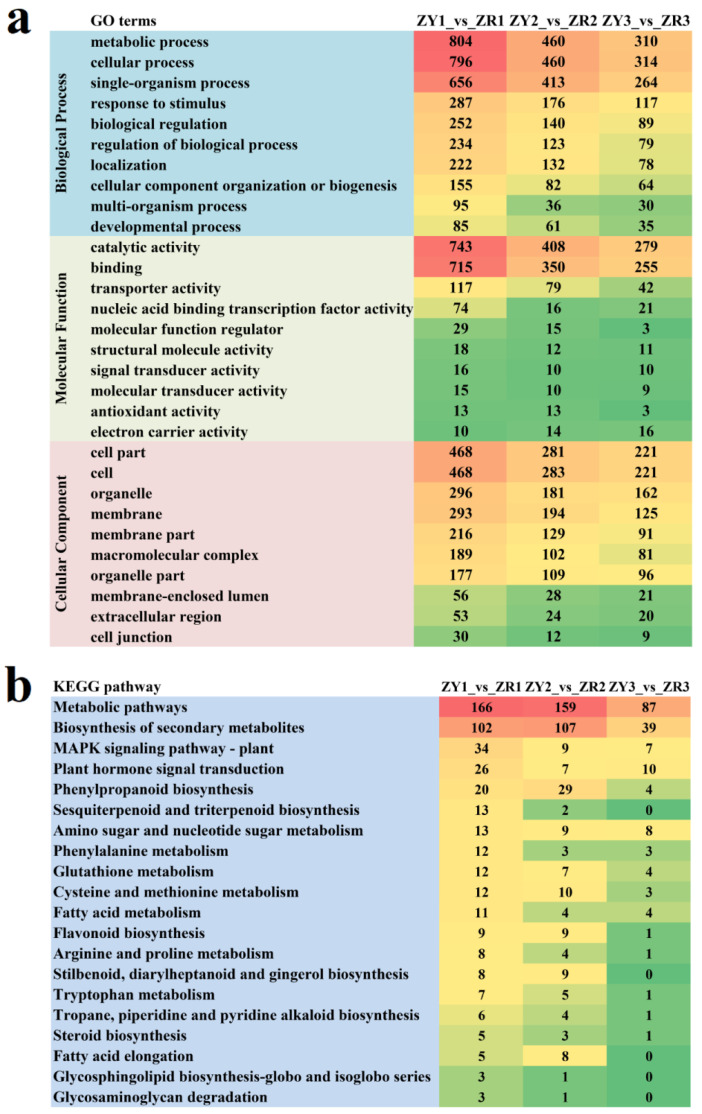
Functional enrichment analysis of DEGs. (**a**) GO enrichment analysis; (**b**) KEGG enrichment analysis.

GO enrichment analysis was used to understand the DEG functions of S1, S2 and S3 using the GO database. These DEGs were classified as molecular functions, biological processes and cellular components, respectively. The top 10 GO terms in each category were examined more closely, and it was determined that metabolic process, cellular process and single-organism process were predominant in the category of biological processes (Table S5–S7). In terms of molecular function, most DEGs were classified into catalytic activity and binding terms, followed by transporter activity. The DEGs in cellular components were mainly enriched in the cell part, cell, organelle, membrane, membrane part, macromolecular complex and organelle part (Figure 2a). 

Functional annotation of the DEGs in the three stages between ZY and ZR was performed based on the KEGG database to further investigate the functions of DEGs that might be related to the formation of pepper fruit color. The results revealed that 108 (Table S8), 98 (Table S9) and 82 (Table S10) pathways were annotated in stages S1, S2 and S3, respectively (Figure 2b).

### 2.4. Variations in Carotenoid Content in the Fruits of ZY and ZR during Fruit Ripening

**Figure 3:**
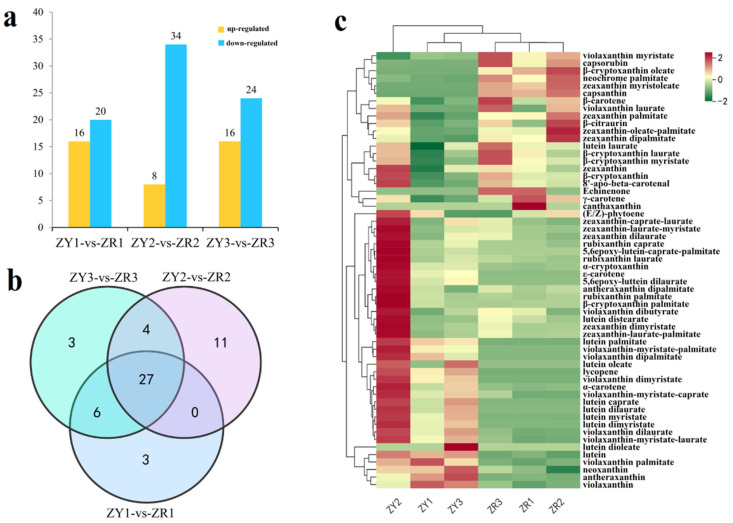
Variations in the carotenoid contents in the pepper fruit of ZY and ZR at three developmental stages. (**a**) The number of upregulated and downregulated DAMs in the pepper at three developmental stages; (**b**) Venn diagram of DAMs in different comparison groups; (**c**) heatmap of carotenoid contents in the pepper fruit of ZY and ZR at three developmental stages.

To investigate the differences in carotenoid components among different-colored pepper fruits, three stages of pepper fruits (S1, S2, and S3) were selected to evaluate the alterations in carotenoid composition and content between ZY and ZR. The targeted metabolomics analysis was performed using UPLC-MS/MS. Differentially expressed metabolites (DAMs) were determined from ZY and ZR at three developmental stages. There were 36 DAMs (16 upregulated and 20 downregulated), 42 DAMs (8 upregulated and 34 downregulated) and 40 DAMs (16 upregulated and 24 downregulated) in stages S1, S2, and S3, respectively (Figure 3a). Of these, 27 DAMs frequently exhibited difference accumulation during the three periods under study (Figure 3b). In total, 58 carotenoid components were identified from the fruits of ZY and ZR, and 54 carotenoid components showed a difference accumulation between ZY and ZR (Figure 3c) (Table S11). 

Most of the individual carotenoids contents in ZY were significantly higher than those in ZR during the three fruit ripening stages. These contents included α-carotene, (E/Z)-phytoene, antheraxanthin, neoxanthin, α-cryptoxanthin, eight violaxanthin individuals and five lutein individuals. For instance, the content of α-carotene in the three development stages of ZY was 13.68μg/g (S1 stage), 65.42μg/g (S2 stage) and 33.45 μg/g (S3 stage), while in ZR, this amount decreased rapidly (to 2.36 μg/g, 3.62μg/g and 4.82μg/g, respectively). There was a higher accumulation of (E/Z)-phytoene content in both ZY and ZR, especially in the first two periods (111.60 μg/g (ZY1), 86.34μg/g (ZR1), 161.45 μg/g (ZY2), and 112.83μg/g (ZR2), respectively). β-carotene, capsorubin, neochrome palmitate, capsanthin, zeaxanthin dipalmitate and zeaxanthin palmitate were highly accumulated in the fruits of ZR. The capsorubin content of capsanthin in the three development stages of ZR was much higher than ZY; however, this content was almost undetectable and could be ignored in the ZY fruits. In addition, the contents of β-cryptoxanthin, β-cryptoxanthin palmitate, Zeaxanthin, four zeaxanthin individuals (zeaxanthin dimyristate, zeaxanthin–laurate–palmitate, zeaxanthin–laurate–myristate, and zeaxanthin dilaurate), and rubixanthin laurate were highest in the ZY2 period, and there is no obvious pattern in other stages between ZY and ZR.

However, several differences in the composition and content of carotenoids were detected in the fruits between ZY and ZR. The accumulation of α-carotene, lutein, antheraxanthin, α-cryptoxanthin and violaxanthin individuals was primarily observed in ZY, while β-carotene, capsorubin, and capsanthin mainly accumulated in ZR.

### 2.5. Identification of WGCNA Modules Associated with Carotenoid Metabolism

**Figure 4:**
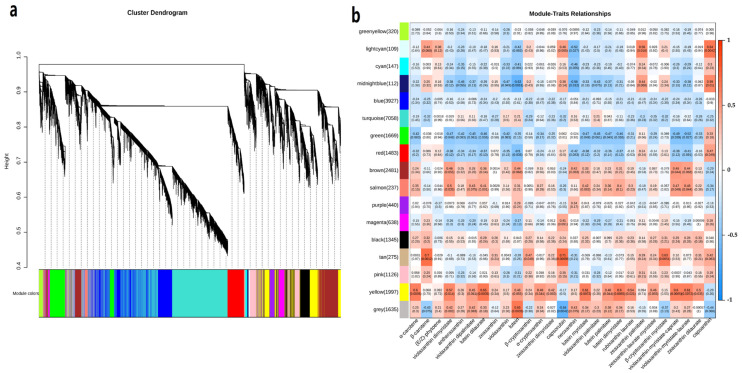
WGCNA of DEGs identified from the fruits of ZY and ZR at three developmental stages. (**a**) Hierarchical cluster tree displaying fourteen modules of co-expressed genes. The lower panel shows modules in specified colors. (**b**) Module–carotenoid weight correlations and corresponding P values (in parentheses). The left panel shows the seventeen modules. The color scale on the right shows module–trait correlation from −1 (blue) to 1 (red).

To characterize the key candidate genes participating in the regulation of carotenoid metabolism in the fruits of ZY and ZR cultivars, weighted gene coexpression network analysis (WGCNA) was implemented to evaluate the associations between DEGs and carotenoid contents of ZY and ZR. The results show that these DEGs could be divided into 17 modules according to their expression patterns (Figure 4a), which are color coded. Based on the above analysis, 27 carotenoid contents, which differed significantly between ZY and ZR, were selected as phenotypic data for the analysis of module–trait correlations. A heatmap was constructed to show the concentration of each phenotypic parameter in each sample; the correlation and corresponding e-value of modules and carotenoids are presented in Figure 4b.

According to the module–trait correlation heatmap, the “tan” module showed a significant positive correlation with β-carotene, capsorubin and β-cryptoxanthin myristate, with correlation coefficients of 0.7, 0.71 and 0.63, respectively, The “tan” module comprised 275 genes, *NCED1*, *AP2-1* (*Capana11g000645*), *ERF039* (*Capana01g000589*) and *CYP71P1* (*Capana05g001955*), which may be involved in carotenoid metabolism. The “yellow” module was significantly positively correlated with α-carotene, lutein dilaurate, lutein myristate, lutein dimyristate, violaxanthin–myristate–caprate and violaxanthin–myristate–laurate, with correlation coefficients of 0.6, 0.65, 0.61, 0.6, 0.6 and 0.61, respectively. The “yellow” module contained 1997 genes, including a number of genes involved in carotenoid metabolism, such as *CYP97C1* (*Capana10g001912*) and *SRG1* (*Capana06g001127*). The “grey” module was significantly positively correlated with lutein, with a correlation coefficient of 0.65. The “lighcyan” module was significantly positively correlated with capsanthin, with a correlation coefficient of 0.64. The “midnightblue” module was significantly negatively correlated with lutein, with a correlation coefficient of −0.62, and the “grey” module was significantly negatively correlated with capsorubin, with a correlation coefficient of −0.64.

### 2.6. Canonical Correlation Analysis

**Figure 5:**
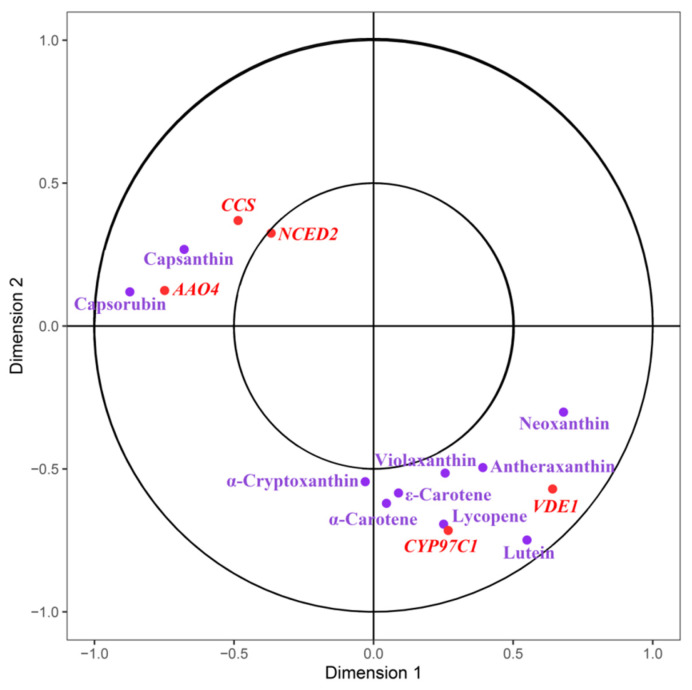
Metabolites of carotenoid metabolism and the CCA of the gene, with metabolite index (purple) and gene ID (red).

The results from transcriptome and metabolome analyses indicate that the structural genes involved in carotenoid biosynthesis exhibit the greatest differential expression during the S2 stage, and there are significant differences in the accumulation of carotenoid metabolites (Figure 5). Thus, the S2 stage may be a critical period for the development of differential pigmentation between ZY and ZR. Although the structural genes and metabolites in the biosynthesis pathway of carotenoids have been largely elucidated, it has yet to be determined which genes play key roles in regulating this process. Therefore, we performed a canonical correlation analysis (CCA) of carotenoid metabolites and gene expression in the related biosynthesis pathways during the S2 stage to identify key candidate genes and their relationships with metabolites. The results indicate that the lutein content is highly correlated with the *VDE1* gene; *CYP97C1* is closely related to the biosynthesis and accumulation of lycopene; while *CCS*, *NCED2* and *AAO4* may be associated with the biosynthesis and accumulation of capsanthin and capsorubin. *VDE1* and *CYP97C1* may also be involved in the biosynthesis and accumulation of other substances such as neoxanthin, antheraxanthin and α-carotene.

### 2.7. Analysis of the Carotenoid Metabolic Pathway in Pepper Fruits

**Figure 6:**
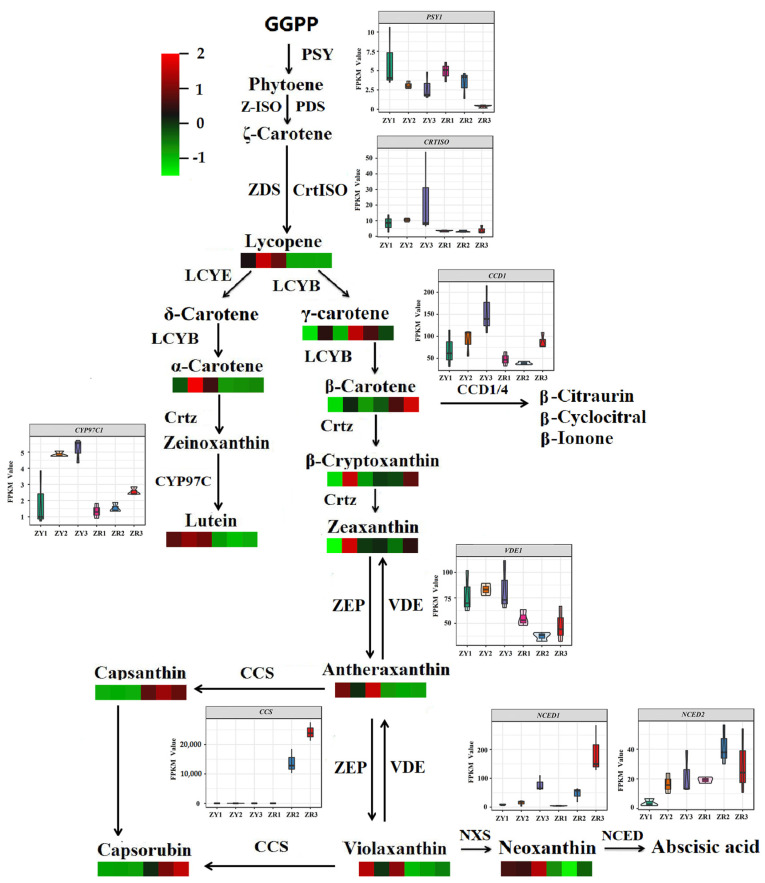
Pathways of carotenoid metabolism in pepper fruits. The carotenoid amounts are shown in heatmaps as ZY1, ZY2, ZY3, ZR1, ZR2, and ZR3 from left to right, respectively.

Compared with ZY and ZR pepper fruits at the same stage, the DEGs involved in the carotenoid metabolism pathway were identified (Figure 6). There were only eight structural genes related to carotenoid biosynthesis between ZY and ZR. The major rate-limiting enzyme *PSY1* showed different expression in the S3 stage, and the expression of *PSY1* was 2.85-fold higher in ZY than in ZR. *CRTISO*, *CCD1*, *CYP97C1* and *VDE1* were upregulated 1.71-fold, 1.22-fold, 1.65-fold, and 1.16-fold in the ZY fruits in the S2 periods, respectively. *NCED2* was downregulated 2.31-fold and 1.32-fold in the ZY fruits in the S1 and S2 periods, respectively. *NCED1* was upregulated 1.26-fold in the ZR fruit at the S3 period. The expression of *CCS* showed the most significant difference: it was upregulated 9.39-fold and 10.00-fold in the ZR fruits in the S2 and S3 periods, respectively. Furthermore, the pepper fruit carotenoid metabolic pathways were mapped. Lycopene, *α*-carotene, lutein, antheraxanthin, violaxanthin and neoxanthin were highly accumulated in the pathway of ZY, while γ-carotene, β-carotene, capsanthin and capsorubin mainly accumulated in ZR and capsanthin and capsorubin were almost non-existent in ZY (Figure 6). Compared with other periods, β-cryptoxanthin and zeaxanthin were synthesized and accumulated in extremely high contents in the S2 period in ZY.

### 2.8. qRT-PCR Analysis

**Figure 7:**
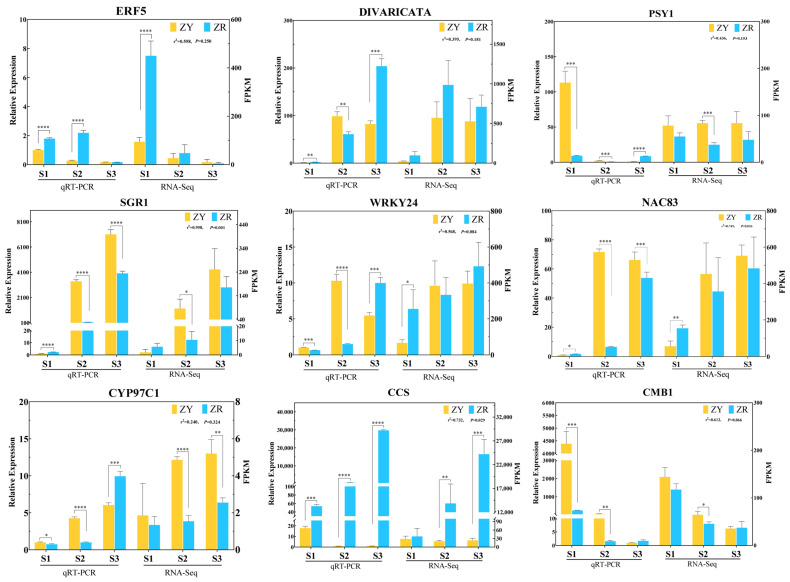
Relative expression of 9 genes involved in carotenoid metabolism during fruit development of ZY and ZR cultivars. All data are presented as the mean of three biological replicates, and error bars represent standard deviation. * indicates that there are significant differences between the two pepper cultivars in the same stages (*p* < 0.05), ** indicates *p* < 0.01, *** indicates *p* < 0.001, **** indicates *p* < 0.0001; the Pearson correlation coefficient is expressed as *r*^2^.

To confirm the accuracy of the RNASeq data, qRT-PCR experiments were performed on carotenoid pathway genes, including *PSY1*, *CYP97C1* and *CCS*, and the transcription factor genes *ERF5*, *DIVARICATA*, *SGR1*, *WRKY24*, *NAC83* and *CMB1*, which may be involved in regulating the carotenoid contents (Figure 7). By comparing the qRT-PCR results and the transcript abundance (FPKM) obtained via transcriptome sequencing, the results showed that the expression levels of the nine genes were basically coincident with the RNA-seq expression data. This supports the reliability of the transcriptome sequencing data in this study.

## 3. Discussion

Fruit color is one of the important phenotypic traits of pepper, which is highly valued by breeders and consumers alike, significantly influencing product choices. One of the main contributors to the rich and diverse variation in peppers’ coloration is the differential accumulation of various types and quantities of carotenoids [12]. Carotenoids can be found in many plants; their accumulation in many flowers, fruits, and roots contributes to their orange, yellow or red coloration and has significant ecological and agronomical value [11]. The synthesis and accumulation of carotenoids in plants such as citrus, tomato and apricot have been studied to establish the mechanism of carotenoid synthesis and accumulation in horticultural crops [3,25,26,28]. Using pepper color mutants as materials that can be used to compare molecular and metabolic levels in the same developmental stage, this study provides new insight into the synthesis and metabolism of pepper carotenoids.

Comparison of the metabolites of ZY and ZR at different developmental stages, it revealed that the number of differential metabolites was highest in the S2 stage (42), followed by S3 (40), and lowest in S1 (36). Not only did the total number of differential metabolites vary among the three stages, but the number of upregulated and downregulated accumulated metabolites also differed. In the S2 stage, 32 differential metabolites were downregulated, while only 8 metabolites were upregulated. We speculate that the S2 period is a crucial time for the formation of color differences between ZY and ZR, during which the upregulated eight types of carotenoid substances (including violaxanthin myristate, zeaxanthin myristoleate, β-cryptoxanthin oleate, zeaxanthin–oleate–palmitate, capsorubin, zeaxanthin dipalmitate, neochrome palmitate, and capsanthin) mainly participate in the formation of ZR fruit color. Of these, capsorubin and capsanthin demonstrated the most significant downregulation, decreasing by 4.92-fold and 7.34-fold, respectively. These compounds have been extensively associated with the development of red pigmentation in pepper fruit [12,29]. We speculated that the significant difference in capsorubin and capsanthin might contribute to the inconsistency in fruit color between ZY and ZR peppers.

In a previous study on the transcriptomics and metabolomics of four types of pepper varieties, it was found that high expression of the *PSY1* gene led to a variation in lycopene content. Moreover, the levels of α-carotene, β-carotene, and γ-carotene in chili peppers were correlated with the expression of *LCYB* and *LCYE* [12]. Research has highlighted a close correlation between the expression levels of the *LCYB* and *LCYE* genes and the content of trans-β-carotene in loquat fruit [30]. Zhou et al. analyzed the transcriptome and metabonomics and found that *PSY*, *NCED1* and *CCD4* were the key genes that informed the significant differences in carotenoid content in the apricot fruits of two cultivars [28]. In this study, the expression levels of *LCYB* and *LCYE* had no significant difference in the S1, S2, and S3 periods of ZY and ZR. The differential expression of *CCD1*, *CYP97C1*, *VDE1*, *CCS*, *NCED1* and *NCED2* may be the main reasons for the differing accumulation of lutein, antheraxanthin, capsorubin, capsanthin, violaxanthin and neoxanthin between the two pepper varieties.

Transcriptional regulation also plays an essential role in carotenoid mechanism. Previous studies have identified ERF, WRKY, NAC, and MYB transcription factors involved in regulating the carotenoid plants, which play a significant role in the formation of fruit color. Overexpression of *SlWRKY35* can increase the accumulation of carotenoids, and targeting *SlWRKY35* can increase the lutein yield in tomato fruits [25]. Twelve WRKY transcription factors were identified in the S1 stage, among which *WRKY24* (*Capana06g001506*) is likely to be involved in the synthesis and regulation of carotenoids. AP2/ERF is an important transcription factor that is closely involved in plant signal transduction and can also be involved in the regulation of plant carotenoid synthesis. Transcription factors such as *CsERF061* and *RAP2.2* are members of the AP2/ERF family. As a transcriptional activator, *CsERF061* can directly bind to the promoter of *LCYb2* and activate the expression of the *LCYb2* gene. *CsERF061* can also activate the expression of key genes such as *PSY1*, *PDS*, *CRTISO*, *LCYb1*, *BCH*, *ZEP*, *NCED3*, *CCD1* and *CCD4*, and can construct a transcriptional regulatory network that promotes the accumulation of carotenoids in citrus fruits [31]. Silencing *RAP2.2* in *Arabidopsis* resulted in significantly downregulated expression of *PSY* and *PDS*, and a nearly 30% reduction in carotenoid content [32]. In this study, it was also found that *RAP2-2* (*Capana02g002706*) was also identified, and *AP2-1* and *ERF039* identified using the WGCAN method may also be involved in regulating the synthesis of some carotenoid substances. Song et al. silenced a MYB-type transcription factor *DIVARICATA1* (*Capana12g002172*), significantly reducing the transcription of the capsaicin synthesis-related genes *PSY*, *PDS*, *β-CH1* and *CCS*, as well as the capsaicin content. In doing so, they demonstrated that *DIVARICATA1* directly binds and regulates the expression of capsaicin synthesis-related genes such as *CCS*. The transcription level of *DIVARICATA1* was positively correlated with capsanthin content [33]. In this study, the differential gene *DIVARICATA* was identified, and its expression pattern was highly similar to *CCS*, and the research results were highly consistent with those of previous studies.

## 4. Materials and Methods

### 4.1. Plant Growth and Sampling

Two pepper (*Capsicum annuum* L.) varieties, ‘ZY’ and ‘ZR’, were provided by College of Horticulture and Landscape, Yunnan Agricultural University (Kunming, China) and grown in a greenhouse (16 h of light at 30 ± 2 °C and 8 h of darkness at 20 ± 2 °C). The ZY variety is a natural mutant derived from a branch mutant of ZR. It was found in Kunming County, Yunnan Province, China, in 2019. ZY and ZR exhibit noticeable differences in fruit color, but no other obvious differences in other traits, and their characteristics have remained stable throughout years of observation and evaluation. The molecular mechanism of color variation in ZY mutant has not been comprehensively studied. The pericarps of fruits in developmental stages S1 (green fruits, 30 days after anthesis (DAA)), S2 (fruit color-changing, 40 DAA) and S3 (maturity, 50 DAA) were collected and pooled from three individual plants, The tissues of ten fruits’ pericarps were sampled, mixed and divided into three parts, quickly frozen in liquid nitrogen and then stored at −80 °C for transcriptome, metabolome analysis and qRT-PCR analysis. 

### 4.2. RNA Extraction, Library Preparation and Sequencing

Total RNA was isolated from ZY and ZR fruit at stages S1, S2, and S3 using the Trizol (Invitrogen, Beijing, China) according to the manufacturer’s protocol. RNA was examined using 1% agarose gels, a NanoPhotometer spectrophotometer (IMPLEN, Westlake Village, CA, USA), a Qubit RNA Assay Kit in Qubit 2.0 Flurometer (Life Technologies, Carlsbad, CA, USA) and a Nano 6000 Assay Kit from the Agilent Bioanalyzer 2100 system (Agilent Technologies, Santa Clara, CA, USA). Next, the high-quality RNA of 18 samples mentioned above was sent to Novogene (Beijing, China) for cDNA library construction. Then, 18 cDNA libraries were sequenced on Illumina Hiseq 4000 platform (paired-end 150 bp), and the raw transcriptome data of samples from ZY and ZR were deposited in the NCBI Short Read Archive (SRA, BioProject ID: PRJNA1030920).

### 4.3. Transcriptome Analysis

Raw data were processed through in-house Perl scripts and adapted accordingly. Low-quality reads were removed using Cutadapt 1.9.1 software (https://cutadapt.readthedocs.io/en/stable/ (accessed on 18 August 2023)), and clean reads were then mapped to the Zunla-1 reference genome (https://www.ncbi.nlm.nih.gov/bioproject/186921 (accessed on 20 August 2023)) using TopHat v2.0.12 [34]. The Cufflinks v2.1.1 Reference Annotation-Based Transcript (RABT) assembly method was used to construct and identify both known and novel transcripts from TopHat alignment results. Next, the FPKM of each gene was calculated based on the length of the gene and the read count mapped to this gene [35]. Differential expression analysis was performed using the DESeq R package (1.18.0) [36]. Log_2_fold change (log_2_FC) value > 1 (upregulated) or <−1 (downregulated) and FDR < 0.05 were considered to be meaningful and used as the significant indigenous boundary for gene expression differences. GO terms, and KEGG pathways analyses were performed using GO seq and KOBAS 3.0 software (http://bioinfo.org/kobas/ (accessed on 21 August 2023)) [37,38], respectively. The terms and pathways with a corrected *p*-value < 0.05 were considered to be significantly enriched. The transcription factor was predicted by using the PlantRegMap/PlantTFDB v5.0 database (http://planttfdb.gao-lab.org/prediction.php (accessed on 22 August 2023)).

### 4.4. Carotenoid Identification and Quantification

The sample was freeze-dried, ground into powder (30 Hz, 1.5 min) and stored at −80 °C until needed. Next, 50 mg powder was weighted and extracted with 0.5 mL mixed solution of n-hexane–acetone–ethanol (1:1:1, *v*/*v*/*v*). The extract was vortexed for 20 min at room temperature. The supernatants were then centrifuged at 12,000 r/min for 5 min at 4 °C prior to collection. The residue was re-extracted by repeating the above steps under the same conditions. After that, it was evaporated to dryness and reconstituted in mixed solution of MeOH/MTBE (1:1, *v*/*v*). The solution was filtered through a 0.22 μm membrane filter for further LC-MS/MS analysis. The carotenoid extracts of pepper fruits were analyzed using an UPLC-APCI-MS/MS system (UPLC, ExionLC™ AD, https://sciex.com.cn/ (accessed on 25 August 2023); MS, Applied Biosystems 6500 Triple Quadrupole, https://sciex.com.cn/ (accessed on 26 August 2023)). The analytical conditions of chromatographic separations were as follows: LC: column, YMC C30 (3 μm, 100 mm × 2.0 mm i.d.); solvent system, methanol: acetonitrile (1:3, *v*/*v*) with 0.01% BHT and 0.1% formic acid (A), methyl tert-butyl ether with 0.01% BHT (B); gradient program, started at 0% B (0–3 min), increased to 70% B (3–5 min), then increased to 95% B (5–9 min), finally ramped back to 0% B (10–11 min); flow rate, 0.8 mL/min; temperature, 28 °C; injection volume for each sample: 2 Μl [12].

Metabolites were detected by using QTRAP^®^ 6500+ LC-MS/MS System, equipped with an APCI (ion source, APCI+; source temperature 350 °C; curtain gas (CUR) were set at 25.0 psi) Heated Nebulizer, operating in positive ion mode and controlled with Analyst 1.6.3 software (Sciex). Carotenoids were analyzed using scheduled multiple reaction monitoring (MRM). Data acquisitions were performed using Analyst 1.6.3 software (Sciex). Multiquant 3.0.3 software (Sciex) was used to quantify all metabolites. Mass spectrometer parameters, including the declustering potentials (DP) and collision energies (CE) for individual MRM transitions, were done with further DP and CE optimization. A specific set of MRM transitions were monitored for each period according to the metabolites eluted within each period. Carotenoids contents were detected using MetWare (http://www.metware.cn/ (accessed on 26 August 2023)) based on the AB Sciex QTRAP 6500 LC-MS/MS platform [12].

### 4.5. Regulatory Network Construction by WGCNA

WGCNA was completed using the Metware Cloud (https://cloud.metware.cn (accessed on 29 August 2023)).

### 4.6. Combined Transcriptomic and Metabolomic Analysis

DAMs were combined with DEGs to analyze the mechanisms underlying the carotenoid synthesis and accumulation of pepper. A canonical correlation analysis (CCA) was used to reflect the overall correlation between the transcriptomes and metabolomes [39].

### 4.7. qRT-PCR Analysis

Total RNA of the three development stages of ZY and ZR was extracted using Trizol reagent and then purified RNA was reversely transcribed into first-strand cDNA using a HiScript II 1st Strand cDNA Synthesis kit. qRT-PCR was performed with the SYBR Green Premix Ex Taq ™ II quantitative PCR system using a Roche LightCycler 480 (Roche, https://www.roche.com (accessed on 30 August 2023)), and each sample had three replications. Relative expressions of target genes were calculated using the 2^−∆∆Ct^ method [40]. All of the primers used for qRT-PCR in this study were listed in Table S12.

## 5. Conclusions

In this study, integrative carotenoid and transcriptome comparisons were carried out at the same developmental stage using two pepper varieties with different fruit colors. The result showed that 93 genes were differentially expressed and 27 carotenoids were differentially accumulated at the three developmental stages. The differential expression of *PSY1*, *CRTISO*, *CCD1*, *CYP97C1*, *VDE1*, *CCS*, *NCED1* and *NCED2* may be the main reasons for the difference accumulation of carotenoids between the two pepper varieties. Numerous transcription factor families of bHLH, MYB, ERF, WRKY, and NAC may be involved in the regulation of carotenoid accumulation. The differing contents of antheraxanthin, capsorubin, capsanthin, violaxanthin and neoxanthin between the two pepper varieties may have a significant influence on the color of the peppers.

## Data Availability

Our RNA-Sseq data were submitted to the Sequence Read Archive (SRA) of NCBI, BioProject ID: PRJNA1030920.

## Supplementary Materials

The supporting information can be downloaded at: https://www.mdpi.com/article/10.3390/ijms242316563/s1.
