# Supplementary material for: Integrative Metabolome and Transcriptome Analyses Provide Insights into Carotenoid Variation in Different-Colored Peppers
Source: Int J Mol Sci. 2023 Nov 21;24(23):16563. doi: 10.3390/ijms242316563 (PMC10706310; doi:10.3390/ijms242316563)
Supplement: Supplementary file 1 [file ijms-24-16563-s001.zip › Table S12.docx]

Table S12 Primer sequences used for real-time qRT-PCR amplification

| **Name** | **Gene ID** | **Primer sequences (5'-3')** | **Primer sequences (5'-3')** |
| --- | --- | --- | --- |
| *ERF5* | *Capana05g001949* | AGGGAGCAAAGCAATCTTGA | ATTTCCACAATCCCAAATCG |
| *DIVARICATA* | *Capana12g002172* | CACGATTCGAAGACAAGCAA | CCATCCAATTCATCCCAATC |
| *GGPPS* | *Capana04g000412* | ATGGCGTTTTCCTGGTTGC | CAATGCGTTATTCCCTTCTCG |
| *PSY1* | *Capana02g002284* | GGTTGGGTTGATGAGTGT | TTGTAGTCATTGGCTTCG |
| *SGR1* | *Capana02g001627* | GTACCCAATTTCTTGATGAA | GCAACAGAATTACTGTCTT |
| *WRKY24* | *Capana06g001506* | TCACCGACCTTCTTGCTTCT | AGAGGAAGGCGAAAAAGGAG |
| *NAC83* | *Capana01g002000* | AATTGTGATGAAGGGCCAAC | TCGGCAGATAACCCAGTTTC |
| *CYP97C1* | *Capana10g001912* | TTGGCAGGCAGCGTTATGTA | TGCCCAGCAACTAGCATTGA |
| *CCS* | *Capana06g000615* | ATGTTATGGCTATTGGTG | TGAGACCCTCTTATCATTC |
| *CMB1* | *Capana10g000710* | ACAACATTTGAACCATCCTGACT | AGGTGCAACGTGAATAGTAGT |
| *UBI3* | *MSTRG.34504* | TTGGCAAGCAACAATCAT | GCAGATGGACAGCAGGAC |
